# Organizational Justice and Perceived Organizational Support: Impact on Negative Work-Home Interference and Well-being Outcomes

**DOI:** 10.5334/pb.bk

**Published:** 2015-10-07

**Authors:** A. Babic, F. Stinglhamber, I. Hansez

**Affiliations:** 1 Human Resources Development Unit, Work Psychology Department, University of Liege, Quartier Agora place des Orateurs, 2 (Bât.32), 4000 Liège, Belgium; 2 Psychological Sciences Research Institute (IPSY), Université catholique de Louvain Place Cardinal Mercier, 10, bte 1348 Louvain-la-Neuve, Belgium

**Keywords:** organizational justice, perceived organizational support, negative work-home interference, well-being

## Abstract

It is well established that negative work-home interference (NegWHI) impacts upon several work attitudes and behaviors. In the interests of both organizational effectiveness and employee well-being, it is important to identify concepts related to NegWHI and investigate their effects on well-being outcomes. This study examines the mediating role of (1) perceived organizational support (POS) in the relationship between organizational justice (OJ) and NegWHI; and (2) NegWHI in the relationships between POS and four well-being outcomes. A total of 509 employees of a Belgian hospital were surveyed. Data were analyzed using structural equation modeling and the bootstrapping method. Results showed that POS partially mediates the relationships between distributive and passive procedural justice and NegWHI, and fully mediates the relationship between the other justice dimensions and NegWHI. NegWHI partially mediates the relationships between POS and both job satisfaction and intention to quit, and fully mediates the relationship between POS and job strain. Furthermore, POS is positively related to job engagement. This study showed that organizations can help employees to better manage their work and family lives and reduce the impact of NegWHI by enhancing employees’ feeling that they are supported by their organization. In order to increase POS, organizations need to promote justice in the workplace.

## Introduction

The increase in dual-career couples and single-parent households means that balancing work and home responsibilities has become more and more difficult (Luk & Shaffer, 2005). When people do not manage to balance the demands of work and family, negative interference between the professional and private spheres may appear, impacting a variety of work-related outcomes such as intention to leave (e.g., Greenhaus, Collins, Singh, & Parasuraman, 1997), job strain (e.g., Judge & Colquit, 2004) and job satisfaction (e.g., Rich, Lepine, & Crawford, 2010).

Among the many factors that may facilitate the balance between work and family responsibilities, a few studies have focused on organizational justice (OJ) (Heponiemi, Elovainio, Pekkarinen, Sinervo, & Kouvonen, 2008; Judge & Colquitt, 2004; Lambert, Hogan, & Cheeseman, 2013). There are several distinct but related justice dimensions, namely distributive (referring to decision outcomes), procedural (referring to the process by which outcomes are reached), interpersonal and informational justice (concerning respectively the quality of interpersonal treatment received, and the information provided during decision making) (Colquitt, 2001). Employee perceptions about the fairness of organizational actions and decisions play a significant role in the development of negative work-home interference (NegWHI) (e.g., Grandey, 2001). Indeed, studies investigating the relationship between OJ and NegWHI highlight that justice at work allows employees to better manage the work-family interface (Heponiemi et al., 2008; Judge & Colquitt, 2004; Lambert et al., 2013).

However, while the effect of OJ on NegWHI has been demonstrated, the theoretical mechanisms underlying this relationship are less clear. If employees perceive fairness at work, why should that reduce their perceptions of NegWHI?

Relationships between OJ and its outcomes are often understood in terms of social exchange. Among theories applying social exchange mechanisms to the employee-organization relationship, the Organizational Support Theory (Eisenberger, Huntington, Hutchison, & Sowa, 1986) considers employees’ favorable reactions to their positive valuations by the organization. According to this theory, perceived organizational support (POS), defined as workers’ perceptions that the organization values their contributions and cares about their well-being (Eisenberger et al., 1986), influences employees’ psychological well-being, the favorableness of their orientation toward the organization and their work, and behavioral outcomes helpful to the organization. POS creates a sense of obligation towards the organization, generates an expectation of reward for increased effort, meets employees’ socio-emotional needs, and produces an expectation that help will be available when needed (Eisenberger & Stinglhamber, 2011).

Considering this framework, fairness in the workplace increases employees’ POS (e.g., Roch & Shanock, 2006). Indeed, fairness at work indicates that employers care about their employees’ well-being (Rhoades & Eisenberger, 2002). By indicating that help is available when needed and by enhancing perceived competences, the feeling of POS increases employees’ capacities to manage work and family demands and therefore reduces their perceptions of NegWHI (e.g., Rhoades & Eisenberger, 2002; Wadsworth & Owens, 2007).

However, to the best of our knowledge, we are not aware of any study investigating social exchange mechanisms in the relationship between OJ and NegWHI. Therefore, the first aim of this study is to examine the OJ-NegWHI relationship from a social exchange perspective by considering POS as one possible mediator.

The effects of POS on a broad range of attitudinal and behavioral outcomes have been largely demonstrated (Rhoades & Eisenberger, 2002). Researchers have attempted to identify the mechanisms underlying these relationships. Research suggests that the effect of POS on intention to quit is mediated through affective (Rhoades, Eisenberger, & Armeli, 2001) and normative commitment (Maertz, Griffeth, Campbell, & Allen, 2007). Self-determined work motivation has been found to partially mediate the POS-job engagement relationship (Gillet, Huart, Colombat, & Fouquereau, 2013). However, to the best of our knowledge, no study has considered NegWHI in the POS-outcomes relationship. Reasoning that feeling supported enhances employees’ ability to manage personal and professional demands, our second objective is to test the mediating role of NegWHI in the relationships between POS and four outcomes (i.e. job satisfaction, job engagement, job strain and intention to quit).

### Organizational Justice and Negative Work-Home Interference: The mediating role of Perceived Organizational Support

The multiple roles in which people have to perform are likely to provoke contradictory demands. In such cases, people experience difficulties in fulfilling the requirements of these multiple roles and perceive interferences among them (Duxbury & Higgins, 1991). The first type of interference, called work-home interference, appears when work demands affect home responsibilities. Conversely, the second type, home-work interference, appears when private life affects work demands (Kossek & Ozeki, 1998). These types of interference constitute work–family conflicts. Considering that the work-to-family direction is more likely to be influenced by an organization’s practices and policies (Friedman & Greenhaus, 2000), in this study, we only focus on negative work-home interference (NegWHI).

NegWHI refers to a form of inter-role conflict in which role pressures from work and home domains are mutually incompatible in some respect. Participation in one role makes simultaneous participation in the other difficult (Greenhaus & Beutell, 1985). An important antecedent of NegWHI is organizational responsiveness to the work-family domain (e.g., Grandey, 2001). Employees’ perceptions of whether their organization is family-supportive negatively predict NegWHI (Allen, 2001). Organizations can take certain actions and apply certain policies to reduce NegWHI, such as flexible schedules or homeworking (e.g., Thomas & Ganster, 1995). One way that organizations can be responsive to work–family concerns is by promoting justice in the workplace notably through work-families policies. According to Grandey (2001), organizations with fair policies and practices are likely to have employees with lower levels of NegWHI. Almost all employees desire fairness and justice in the workplace (Greenberg & Folger, 1983).

The construct of organizational justice (OJ) generally refers to four specific components (Colquitt, 2001). *Distributive justice* refers to employees’ perception that fairness exists in the allocation of rewards (Folger & Cropanzano, 1998). According to equity theory (Adams, 1965), workers evaluate the fairness of organizational outputs by examining the ratio between received outcomes and their contributions to the organization. Workers also consider rewards distributions to be fair when they perceive equitable distribution of resources in the organization to peers or other employees whose jobs are comparable to theirs (Leventhal, 1976). *Procedural justice* refers to employees’ perception of the fairness of decision-making procedures concerning any item of value that the organization provides. Procedures are considered fair if they allow employees to give their own opinions for decision making (Thibaut & Walker, 1975), eliminate bias, allocate resources consistently, use accurate information, can be corrected if decisions made turn out to be poor, represent the concerns of all those affected by decisions, and reflect prevailing norms of moral and ethical standards (Leventhal, 1980). *Interpersonal justice* refers to employees’ perception concerning the fairness of treatment received from the organization and managers (i.e., proper and respectful treatment, Greenberg, 1993). *Informational justice* defines the extent to which explanations given are compatible with the decisions reached (e.g., why certain outcomes have been distributed in a certain way). Employees also judge whether they receive adequate explanations in a timely manner (Greenberg, 1993).

The few studies focusing on OJ and NegWHI have pointed out a negative association between these concepts. Adapting justice items to work-family policies, decisions and procedures, Judge and Colquitt (2004) found that procedural justice was negatively related to NegWHI. By considering employees’ opinions and establishing consistent, unbiased, accurate and ethical work–family procedures, organizations show that they are concerned about work–family balance (Leventhal, 1980), thereby decreasing employees’ perceptions of NegWHI.

These authors also found a negative relationship between interpersonal justice and NegWHI. More recently, Heponiemi et al. (2008) found that interactional justice (i.e., a combination of interpersonal and informational justice, Bies & Moag, 1986) was negatively related to NegWHI. Being understood and treated with dignity and respect by authorities reduces employees’ perceptions of NegWHI. When working in a workplace applying fair work-family policies, employees know that their employer will be understanding and offer fair treatment and support if for some reason, family issues need more attention, thus decreasing their perception of NegWHI.

Using data from two different correctional facilities (i.e., 272 employees from public prison staff; 160 employees from private prison staff), Lambert et al. (2013) found that low distributive justice generates NegWHI. When organizational outcomes are perceived as being unjust, the person-environment fit decreases. This misfit generates negative feelings which can spill over into home life, increasing the perception of NegWHI (Lambert, 2003).

While a few studies have thus shown that justice dimensions are related to NegWHI, the theoretical mechanisms underlying those effects are less clear. For some scholars, fair treatment increases employees’ self-esteem (Tyler & Blader, 2000) and the trust that employees have towards their organization (e.g., Lind & Van den Bos, 2002), leading them to better cope with difficult or uncertain situations. According to the instrumental model (Thibaut & Walker, 1975), the uncertainty management theory (Lind & Van den Bos, 2002) or the fairness heuristic theory (Van den Bos, Lind, & Wilke, 2001), justice has the ability to reduce employees’ feelings of lack of control and uncertainty. By reducing those feelings, fairness enhances employees’ abilities to juggle the role demands of their personal and professional lives (Judge & Colquitt, 2004).

Others have argued that individuals working in a fair workplace perceive less NegWHI because fair organizations are more responsive to work–family issues (Grandey, 2001) and more supportive towards their employees (Allen, 2001). The perceived support is thus supposed to have an important role in this relationship. However, we are not aware of any study investigating the role of support in the OJ-NegWHI relationship. Considering the Organizational Support Theory (Eisenberger et al., 1986), we think it is relevant to consider perceived organizational support (POS) as one possible mediator. POS has been defined in terms of the quality of the relationship between employees and their organization. Specifically, it refers to “employees’ global beliefs concerning the extent to which their organization values their contributions and cares about their well-being” (Eisenberger, Huntington, Hutchison, & Sowa, 1986, p. 501).

According to Rhoades and Eisenberger (2002), OJ is one of the strongest antecedents of POS because employees view fair organizational treatment as an indication that their organization cares about them. Fasolo (1995) claimed that allowing employees to have a voice in decision-making procedures indicates that the organization is concerned about and cares for its employees. Fair procedures also imply that the organization respects employees’ rights, and this contributes positively to POS (Moideenkutty, Blau, Kumar, & Nalakath, 2001). To determine whether their organization values them, employees also pay attention to the fairness of outcomes received from the organization. Fasolo (1995) and Loi, Hang-yue and Foley (2006) found that both fair procedures (i.e., procedural justice) and fair pay (i.e., distributive justice) were strongly related to POS. Distributive justice leads employees to make inferences regarding the willingness of their organization to reward or help them. Such inferences result in perceptions that their organization values their contributions. In her study, Cheung (2013) found that interpersonal and informational justices were also positively related to POS. Being treated with dignity and respect in the administration of outcomes and receiving adequate information about these both create a strong feeling that the organization values one’s contribution and cares about one’s well-being.

Regarding the consequences of POS, the Organizational Support Theory (Eisenberger et al., 1986) suggests two possible ways in which POS could affect NegWHI. Firstly, POS indicates that emotional and material resources or help are available when needed to allow employees to carry out their job more effectively (Rhoades & Eisenberger, 2002). The more employees feel supported, the more they know that help is available and the less they perceive NegWHI. Secondly, POS may decrease employees’ perception of NegWHI by enhancing perceived competence (i.e., self-efficacy) (Rhoades & Eisenberger, 2002). Support enhances a sense of being able to cope effectively with the demands of various roles which, in turn, reduces the experience of NegWHI. Many studies have examined the influence of POS on work-family conflict. Based on a sample of 341 public organization employees, Wadsworth and Owens (2007) found that POS reduced the level of NegWHI. Meta-analyses have shown that work support (including organizational support) is related to work-home interference (e.g., Byron, 2005; Michel, Kotrba, Mitchelson, Clark, & Baltes, 2011).

Considering that fairness contributes to employees’ assessment of organizational support and that perceiving support reduces the perception of NegWHI, our first hypothesis predicts that:

*Hypothesis 1:* POS will mediate the negative relationship between the four OJ dimensions and NegWHI.

### Perceived Organizational Support and well-being outcomes: The mediating role of Negative Work-Home Interference

Work-family conflict has become a major attribute of human life today and is associated with many health, well-being, and organizational outcomes. In their meta-analysis, Allen, Herst, Bruck, and Sutton (2000) identified three categories of outcomes: stress-related, work-related and non-work-related outcomes. More recently, the meta-analysis of Amstad, Meier, Fasel, Elfering, and Semmer (2011) also identified three categories: work-related, family-related and domain-unspecific outcomes.

Work stress is the strongest outcome related to NegWHI among stress-related (Allen et al., 2000) and work-related outcomes (Amstad et al., 2011). In their cross-sectional study of 422 firemen in Taiwan, Wong, Lin, Liu, and Wan (2014) found that NegWHI was positively related to job strain. This result may be explained by the Scarcity Hypothesis (Marks, 1977; Sieber, 1974) which states that people have finite personal resources (i.e., time, energy, and attention). As these resources are scarce, they have to be allocated effectively among the different roles an individual occupies. However, in line with the Resource Drain Theory (Edwards & Rothbard, 2000), individuals inevitably experience substantial resource drain that compromises their effectiveness in their multiple roles. Indeed, using resources to fulfill role obligations in one domain reduces available resources that could be used to fulfill role obligations in the other domain. Therefore, individuals engaged in numerous roles may reduce the resources available to fulfill all their role demands, thereby causing role conflict between work and family (Goode, 1960) which may contribute to the experience of stress or strain (Casper, Martin, Buffardi, & Erdwins, 2002). Thus, enacting multiple roles can drain individuals’ resources, impacting role enactment negatively and causing stress (Aryee, Srinivas, & Tan, 2005).

Intention to quit displays the strongest relationship with NegWHI among work-related outcomes (Allen et al., 2000). Results of Amstad et al. (2011) also showed that turnover intention is significantly related to NegWHI. According to the Conservation of Resources Theory (Hobfoll, 1989), people are motivated to acquire, preserve, protect and expand their resources in order to deal with the demands of work and family. When people perceive a (threat of) loss of resources and when no action is taken, a loss spiral appears in which more and more resources are lost. When resources are lost or threatened, people perceive work-family conflict. In order to preserve and protect their resources, people perceiving that their work interferes negatively with their family want to flee this situation (Hobfoll, 1989) and therefore have greater intention to quit the organization (e.g., Greenhaus et al., 1997).

Job satisfaction appears to be the work-related outcome that has attracted the most research attention (Allen et al., 2000; Amstad et al., 2011). When work causes difficulties in fulfilling family responsibilities, the individual gets lower satisfaction from work (e.g., Höge, 2009). Indeed, NegWHI has been associated with diminished value attainment, which itself has been associated with low levels of job satisfaction (Perrewe, Hochwarter, & Kiewitz, 1999).

Empirical research has shown that the work-family conflict that employees have to deal with can impact their work engagement (e.g., Fiksenbaum, 2014; Wilczek-Ruzyczka, Basinska, & Dåderman, 2012). Based on a sample of 98 nurses from southern Poland, Wilczek-Ruzyczka et al. (2012) found that a group of nurses with higher NegWHI was characterized by less vigor and less dedication than the group with lower NegWHI. Perceiving that work interferes strongly and negatively with family drains individuals’ resources, leading them to engage themselves less in their work in order to protect their remaining resources (Hobfoll, 1989). Fiksenbaum (2014) found that, in a supportive culture, the more NegWHI decreases, the more individuals are likely to have the energy to invest in their work. To reciprocate their feelings of support and demonstrate their dedication toward the organization, employees may want to engage more in work activities.

If NegWHI is related to the well-being outcomes included in the present study, POS also appears to be related to these. According to the Organizational Support Theory (Eisenberger et al., 1986), POS should meet workers’ expectations that the organization will provide sympathetic understanding and material aid to deal with stressful situations and will consequently meet employees’ need for emotional support. Therefore, the more workers feel supported, the more they expect that emotional and tangible resources will be available if needed and the more they feel high job satisfaction and reduced job strain (e.g., Gillet, Colombat, Michinov, Pronost, & Fouquereau, 2013).

POS serves as an important component in the social exchange relationship between employees and the organization by eliciting a sense of obligation to return the favorable treatment one has received (Eisenberger & Stinglhamber, 2011). The norm of reciprocity (Gouldner, 1960) dictates the return of (un)favorable treatment to the donor. Well-treated workers feel obliged to return the favorable treatment received by helping their organization to fulfill its objectives. The more workers feel supported, the more they are motivated to be positively oriented toward their organization and to express stronger feelings of affiliation and loyalty to their organization. Therefore, when perceiving support, employees have less intention to quit their organization (e.g., Dawley, Houghton, & Bucklew, 2010).

POS also increases intrinsic task interest (Eisenberger & Stinglhamber, 2011) leading workers to be more engaged in their work (Gillet, Huart, et al., 2013). Indeed, the expectation that resources will be provided when needed and that high performance will be rewarded increase intrinsic task interest. Receiving positive feedback also meets employees’ needs for approval and self-esteem, leading to increased intrinsic interest. In the same vein, the self-efficacy resulting from POS, makes work more interesting (Caesens & Stinglhamber, 2014).

There is thus good evidence to assume that both NegWHI and POS are related to the four outcomes included in the present study. By enhancing perceived competence and knowing that help is available when needed, POS also decreases the perception of NegWHI (e.g., McManus, Korabik, Rosin, & Kelloway, 2002). Based on these elements, our second hypothesis posits that NegWHI mediates the relationship between POS and the four work-related outcomes (i.e., job strain, intention to quit, job satisfaction and job engagement).

*Hypothesis 2:* NegWHI will mediate the effects of POS on (a) job strain, (b) intention to quit, (c) job satisfaction and (d) job engagement.

**Figure 1:**
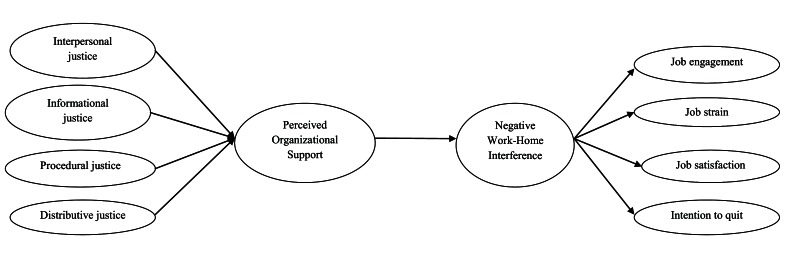
Hypothesized theoretical model.

Figure 1 depicts the hypothesized theoretical model. As shown in this figure, POS is assumed to mediate the relationship between the four dimensions of OJ and NegWHI. In addition, NegWHI is assumed to mediate the link between POS and work-related outcomes.

## Method

### Sample and procedure

In order to test our hypotheses, a self-reported questionnaire was administered to employees in the “Care Center” of a Belgian hospital. This organization has implemented several work-family policies including the possibility to organize working time (the most used) (e.g., flexible schedule, early-retirement arrangements), various types of days off (e.g., for palliative care), or other kinds of support (e.g., assistance with payment of medical fees, company nursery). After informative meetings with management, the personnel department and head nurses, employees received a paper and pencil questionnaire in the workplace. A letter explaining the goal of the study was attached to the questionnaire. One thousand seven hundred ninety-eight people were invited to participate in this research. People were given two months to complete the questionnaires which were anonymous and confidential. They were collected using sealed ballot boxes. We received 509 questionnaires in return, corresponding to a response rate of about 28%.

Most of the participants were female (85%) and employed full-time (59%). About 73% worked in a care job (e.g., nurse, child care worker), 13% worked in a technical job (e.g., logistics assistant, medical laboratory technician), 9% worked as paramedical staff (e.g., social worker, psychologist) and 5% worked as administrative personnel (e.g., medical secretary, executive secretary). Sixty-five percent of the participants had children (22% had one child, 31% had two and 12% had three or more). Nineteen percent of them had preschool children (0–5 years old), fifteen percent had elementary school children (6–12 years old), fourteen percent had children from 13 to 18 years old and finally 16% had children of 19 years old or more. Seventy-four percent of the respondents were either married or living with a partner. Among participants, 63% had a spouse working full-time. About 34% of respondents were between 25 and 35 years old. They had been employed by their company on average for 12.90 years (*SD* = 9.19) and had been working within their department on average for 9.52 years (*SD* = 8.34). Forty-five percent of the participants worked overtime.

Using the full partial covariate effects (Little, 2013), seven socio-demographic variables (age, organizational and departmental tenure, overtime, number of children, presence of children of 6–12 years old and rhythm of work) were significantly related with the constructs of our model. Consequently, we included these seven socio-demographic variables as covariates to control for their effects in our analyses.

### Measures

*Organizational Justice* (OJ) was measured using the scale developed by Judge and Colquitt (2004). These authors adapted the four-dimensional measure created and validated by Colquitt (2001) to work-family balance, with items referring to work-family policies, decisions and procedures. Procedural justice was measured with seven items (e.g., “I had some influence on the final result of work-family policies”); distributive justice with four items (e.g., “The availability of work-family assistance is appropriate for the work I have completed”); interpersonal justice with four items referring to the person carrying out or applying work-family policies (e.g., “He or she treats me politely”); and informational justice with five items (e.g., “He or she explains work-family policies and issues thoroughly”). People responded on a 5-point Likert-type scale (1: strongly disagree to 5: strongly agree).

*Perceived Organizational Support* (POS) was measured using Eisenberger, Cummings, Armeli and Lynch’s (1997) short version of the Survey of Perceived Organizational Support (Eisenberger et al., 1986). This short version included eight items (e.g., “My organization really cares about my well-being”). People responded on a 5-point Likert-type scale (1: strongly disagree to 5: strongly agree).

*Negative Work-Home Interference* (NegWHI) was measured using the appropriate SWING subscale (Geurts et al., 2005). This nine-item subscale evaluates the negative impact of the professional situation on family life (e.g., “I’m irritable at home because my work is demanding”). People responded on a 4-point Likert-type scale (0: never to 3: always).

*Job strain* was measured with the Negative Occupational State Inventory subscale developed by Barbier, Peters and Hansez (2009) which has been used in diverse occupational fields (e.g., Hansez & Chmiel, 2010). This scale comprises nine items (e.g., “My work stresses me”). People responded on a 4-point Likert-type scale (1: never to 4: always).

*Job engagement* was measured with the Positive Occupational State Inventory subscale developed by Barbier et al. (2009) which has also been used in diverse fields (e.g., Hansez & Chmiel, 2010). This scale included eight items (e.g., “When I’m working I forget my tiredness”). People responded on a 4-point Likert-type scale (1: never to 4: always).

*Job satisfaction* was measured with the scale used by Eisenberger, Cummings, Armeli, and Lynch (1997). This scale comprised four items (e.g., “All in all, I am very satisfied with my current job”). People responded on a 5-point Likert-type scale (1: strongly disagree to 5: strongly agree).

*Intention to quit* was estimated using Hom and Griffeth’s (1991) scale which comprises three items (e.g., “I often think about quitting my organization”). People responded on a 5-point Likert-type scale (1: strongly disagree to 5: strongly agree).

### Data analyses

Structural equation modeling analyses were performed using Lisrel 8.80 (Jöreskog & Sörbom, 2006). Data were analyzed following a two-stage process suggested by Anderson and Gerbing (1988). First, we assessed the measurement model through a series of confirmatory factor analyses to evaluate the independence of constructs examined in our study. Second, we proceeded with the assessment of the hypothesized structural relationships among latent variables. For this second stage, in order to limit the number of parameters to be estimated, we reduced the number of items per factor by combining them to create a limited number of indicators per construct (Landis, Beal, & Tesluk, 2000). Using the balancing technique, we generated aggregate indicators by averaging items with high and low loadings. We thus reduced the number of items to three for each of our constructs. This parceling strategy preserves common construct variance while minimizing unrelated specific variance (e.g., Little, Cunningham, Shahar & Widaman, 2002).

## Results

### Discriminant validity

We tested the distinctiveness between the variables included in our study by comparing several nested models (Bentler & Bonett, 1980). First, we examined the fit of our hypothesized ten-factor model (i.e., procedural, distributive, interpersonal and informational justice, POS, NegWHI, job strain, job engagement, job satisfaction and intention to quit). The results indicate that this hypothesized measurement model fitted the data reasonably well (χ2(450) = 1736.50, *p* < .001, RMSEA = .08, NNFI = .94, CFI = .95). However, the loadings of three procedural justice items were under .50, which is the recommended cutoff score for factor loadings (Kline, 2011). We also tested 3 nine-factor models and 2 eight-factor models obtained by combining several dimensions of OJ. However, these five more constrained measurement models did not fit the data well.

In their study, Jepsen and Rodwell (2009) investigated the dimensionality of the Colquitt justice measures (Colquitt, 2001) across a wide range of service occupations. Using confirmatory factor analyses, they found an additional fifth factor included in the initial procedural justice dimension. Among the seven items evaluating procedural justice, the authors allowed three to load on this new latent variable. This fifth factor, named “procedural voice” by the authors, assesses employees’ possibility to take an active part in the decision-making process by expressing their views and feelings about organizational procedures and by influencing them. The remaining four items loaded on the factor named “procedural” by the authors. These items evaluate employees’ perception concerning the fairness of decision-making procedures by referring to the intrinsic quality of those procedures (i.e., procedures without bias, applied consistently, based on accurate information and respecting ethical and moral criteria). This factor has its origins in the characteristics determining fair procedures (Leventhal, 1976, 1980). The authors showed that the five-factor model (i.e., procedural voice, procedural, distributive, informational and interpersonal justice) displayed a significantly better fit than Colquitt’s four-factor model of justice.

Therefore, we tested an eleven-factor model by integrating Jepsen and Rodwell’s results. To clarify the distinction between the sub-dimensions of procedural justice, we changed their names. We renamed “procedural voice” as “active form of procedural justice”, and renamed the second sub-dimension (simply named “procedural” by Jepsen and Rodwell) “passive form of procedural justice”. Our items’ distribution between these two sub-dimensions of procedural justice was the same as that of Jepsen and Rodwell. Three items loaded on the active form (i.e., item 1 “I had some influence on the final result of work-family policies”; item 2 “I have been able to express my views and feelings during the implementation of work-family policies”; and item 3 “I had the possibility to contest the final result of work-family policies”). The other four items loaded on the passive form (i.e., item 4 “Work–family policies have been based on accurate information”; item 5 “Work-family policies are applied consistently”; item 6 “Work-family policies have been free of bias”; and item 7 “Work-family policies respect ethical and moral standards”). This eleven-factor model fitted the data well (χ2(440) = 1071.14, *p* < .001, RMSEA = .05, NNFI = .97) and was significantly superior to the hypothesized ten-factor model (Δχ²(10) = 665.36, *p* < .001). All loadings were higher than .50.

Starting from this eleven-factor model, we tested a series of more constrained measurement models. Chi-square difference tests were used to compare the fit of these nested models with that of the eleven-factor model (Bentler & Bonett, 1980). Results indicate that the eleven-factor model was significantly superior to all alternative models. Consequently, we used this eleven-factor model to test our hypotheses. Table 1 displays fit indices for some of these alternative models.

**Table 1:** Fit indices for measurement models. *Note. N* = 509. Jppa = passive procedural justice; Jpac = active procedural justice; Jdis = distributive justice; Jinter = interpersonal justice; Jinfo = informational justice; χ^2^ = Minimum Fit Function Chi-Square; df = degrees of freedom; NNFI = Non-Normed Fit Index; CFI = Comparative Fit Index; RMSEA = Root-Mean-Square Error of Approximation; Δχ^2^ = chi-square difference tests between the eleven-factor model and alternative models. ***p < .001.

Models		χ^2^	df	χ^2^ / df	NNFI	CFI	RMSEA	Δχ^2^ (Δdf)	Model comparisons

1	11-factor model	1071.14***	440	2.43	.97	.98	.05	––	––
2	10-factor model : Jpac with Jppa	1736.50***	450	3.86	.94	.95	.08	665.36(10)***	1 VS 2
3	10-factor model : Jpac with Jdis	2141.46***	450	4.76	.93	.94	.09	1070.32(10)***	1 VS 3
4	10-factor model : Jpac with Jinter	2807.16***	450	6.24	.91	.92	.10	1736.02(10)***	1 VS 4
5	10-factor model : Jpac with Jinfo	2273.44***	450	5.05	.93	.94	.010	1202.30(10)***	1 VS 5
6	10-factor model : Jppa with Jdis	1430.25***	450	3.18	.96	.97	.07	359.11(10)***	1 VS 6
7	10-factor model : Jppa with Jinter	1739.61***	450	3.86	.95	.96	.08	668.47(10)***	1 VS 7
8	10-factor model : Jppa with Jinfo	1568.36***	450	3.48	.95	.96	.07	497.22(10)***	1 VS 8
9	10-factor model : Jdis with Jinter	2658.59***	450	5.91	.91	.93	.11	1587.45(10)***	1 VS 9
10	10-factor model : Jdis with Jinfo	2015.60***	450	4.48	.94	.95	.09	944.46(10)***	1 VS 10
11	10-factor model : Jinter with Jinfo	1259.75***	450	2.80	.97	.97	.06	188.61(10)***	1 VS 11

### Relationships among variables

Means, standard deviations, Cronbach’s alphas and correlations among variables are presented in Table 2. Internal consistency reliabilities ranged from .78 to .92. On average, participants perceived a low level of OJ (means ranged from 2.19 to 3.40 with *SD* from .71 to .93, range = 1–5) and a rather low POS (*M* = 2.56, *SD* = .64, range = 1–5). On average, they sometimes perceived NegWHI (*M* = 1.01, *SD* = .60, range = 0–3). They were somewhat satisfied with their job (*M* = 3.52, *SD* = .90, range = 1–5) and showed low levels of intention to leave their organization (*M* = 2.16, *SD* = .98, range = 1–5). They sometimes felt strained by their job (*M* = 1.80, *SD* = .52, range = 1–4) and were engaged in their work (*M* = 2.80, *SD* = .48, range = 1–4).

**Table 2:** Descriptive statistics and inter-correlations among variables. *Note. N* = 509. POS = perceived organizational support; NegWHI = negative work-home interference. Correlations among variables are provided below the diagonal and Cronbach’s alphas are provided on the diagonal. *p < .05. **p < .01. ***p < .001. Absence of means and standard deviations for rhythm of work, age, presence of children between 6–12 years old, and overtime because the answers were beforehand categorized in the questionnaire.

	Variables	M	SD	1	2	3	4	5	6	7	8	9	10	11	12	13	14	15	16	17	18

1	Rhythm of work	–	–	–																	
2	Age	–	–	−.11*	–																
3	Number of children	1.21	1.05	−.28***	.40***	–															
4	Children of 6–12 years old	–	–	−.06	.02	.34***	–														
5	Organizational tenure	12.90	9.19	−.11*	.78***	.39***	.12**	–													
6	Tenure in the department	9.52	8.35	−.04	.60***	.24***	.05	.76***	–												
7	Overtime	–	–	.04	−.02	−.04	−.04	.08	.05	–											
8	Active procedural justice ^1^	2.19	.93	.01	−.03	−.08	−.07	-.02	−.13**	−.04	(.91)										
9	Passive procedural justice ^1^	2.84	.71	−.04	−.07	−.01	−.13**	-.08	−.08	−.10*	.38***	(.86)									
10	Distributive justice ^1^	2.65	.78	−.07	−.07	.02	−.06	-.07	−.15**	−.15***	.43***	.58***	(.92)								
11	Interpersonal justice ^1^	3.40	.73	−.07	−.06	.07	.02	-.05	−.09	−.10*	.25***	.40***	.35***	(.92)							
12	Informational justice ^1^	3.06	.71	−.02	−.05	.00	−.01	-.05	−.07	−.16***	.34***	.48***	.49***	.78***	(.90)						
13	POS^1^	2.56	.64	−.06	−.05	−.02	−.03	-.07	−.14**	−.16***	.35***	.45***	.46***	.43***	.49***	(.86)					
14	NegWHI^2^	1.01	.60	.14**	−.06	−.11**	−.06	-.01	−.01	.33***	−.11*	−.34***	−.37***	−.27***	−.29***	−.35***	(.90)				
15	Job engagement^3^	2.80	.48	−.03	−.05	.01	−.05	-.10*	−.07	.01	.10*	.17***	.07	.20***	.19***	.25***	−.13**	(.78)			
16	Job strain^3^	1.80	.52	.06	−.04	−.14**	−.07	.02	.04	.21***	−.15***	−.30***	−.32***	−.28***	−.29***	−.31***	.68***	−.23***	(.86)		
17	Job satisfaction^1^	3.52	.90	−.03	−.05	.06	.02	-.06	−.07	−.18***	.20***	.34***	.33***	.32***	.36***	.40***	−.49***	.47***	−.56***	(.89)	
18	Intention to quit^1^	2.16	.98	.05	−.19***	−.18***	−.07	-.15***	−.07	.20***	−.13**	−.21***	−.23***	−.28***	−.29***	−.33***	.43***	−.25***	.45***	−.57***	(.91)

^1^ Ranges from 1 (strongly disagree) to 5 (strongly agree). ^2^ Ranges from 0 (never) to 3 (always). ^3^ Ranges from 1 (never) to 4 (always).

Based on the results of the confirmatory factor analyses, we examined the structural relationships among latent variables through a series of alternative models (Models 2 to 10). Table 3 presents the fit indices for these alternative models. In all models, the disturbance terms of our four outcomes were allowed to correlate. Model 1 (i.e., the hypothesized model) fitted the data reasonably well (χ2(690) = 2427.42, *p* < .001, RMSEA = .07, NNFI = .94, CFI = .95).

**Table 3:** Fit indices for structural models. *Note. N* = 509. Jppa = passive procedural justice; Jpac = active procedural justice; Jdis = distributive justice; Jinter = interpersonal justice; Jinfo = informational justice; POS = perceived organizational support; NegWHI = negative work-home interference; OJ = organizational justice; χ^2^ = Minimum Fit Function Chi-Square; df = degrees of freedom; NNFI = Non-Normed Fit Index; CFI = Comparative Fit Index; RMSEA = Root-Mean-Square Error of Approximation. ***p < .001.

Models	χ^2^	df	χ^2^ / df	NNFI	CFI	RMSEA	Δχ^2^ (Δdf)	Model comparisons

Model 1: theoretical model (with Jpac and Jppa separated)	2427.42	690	3.52	.94	.95	.07	–	–
Model 2: Model 1 + Path between POS and job satisfaction	2411.16	689	3.50	.94	.95	.07	16.26(1)***	Model 1 vs Model 2
Model 3: Model 2 + Path between POS and job engagement	2399.33	688	3.49	.94	.95	.07	11.83(1)***	Model 2 vs Model 3
Model 4: Model 3 + Path between POS and intention to quit	2372.30	687	3.45	.94	.95	.07	27.03(1)***	Model 3 vs Model 4
Model 5: Model 4 + Path between POS and job strain	2368.66	686	3.45	.94	.95	.07	3.64(1)	Model 4 vs Model 5
Model 6: Model 4 + Path between Jppa and NegWHI	2360.21	686	3.44	.94	.95	.07	12.09(1)***	Model 4 vs Model 6
Model 7: Model 6 + Path between Jdis and NegWHI	2346.99	685	3.43	.94	.95	.07	13.22(1)***	Model 6 vs Model 7
Model 8: Model 7 + Path between Jppac and NegWHI	2344.08	684	3.43	.94	.95	.07	2.91(1)	Model 7 vs Model 8
Model 9: Model 7 + Path between Jinter and NegWHI	2345.48	684	3.43	.94	.95	.07	1.51(1)	Model 7 vs Model 9
Model 10: Model 7 + Path between Jinfo and NegWHI	2346.25	684	3.43	.94	.95	.07	.74(1)	Model 7 vs Model 10

To evaluate whether this model offered the best depiction of our data, we successively added paths from POS to job satisfaction (Model 2), to job engagement (Model 3), to intention to quit (Model 4). Model 4 presented a fit that was superior to preceding models. We added a path from POS to job strain (Model 5), but Model 5 did not have a significantly better fit than Model 4 (Δχ²(1) = 3.64, *p* > .05).

Starting with Model 4, we successively added paths from passive procedural justice to NegWHI (Model 6) and from distributive justice to NegWHI (Model 7). Model 7 presented a fit that was superior to Model 4 and Model 6. We also added paths from active procedural justice (Model 8), interpersonal justice (Model 9), and informational justice (Model 10) to NegWHI but these three latter models did not have a significantly better fit than Model 7.

**Figure 2:**
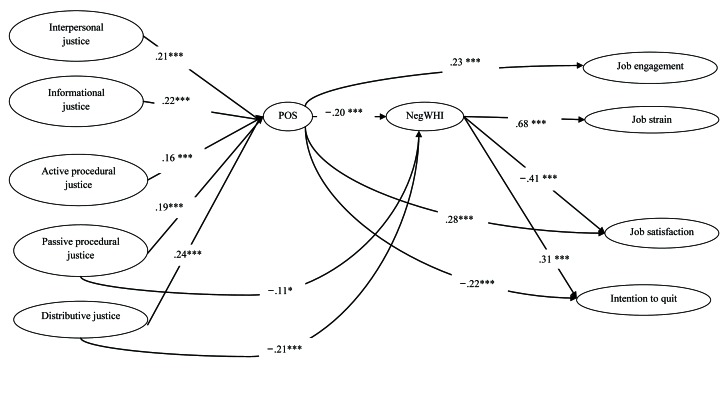
Completely standardized path coefficients for the retained model (Model 7). For the sake of clarity, only structural relationships are shown. POS = perceived organizational support; NegWHI = negative work-home interference; *p < .05; **p < .01; ***p < .001.

Standardized parameter estimates for this Model 7 are shown in Figure 2. For ease of presentation, we show the structural model rather than the full measurement model. Regarding our first hypothesis, all five dimensions of OJ were positively associated to POS which, in turn, was significantly and negatively associated with NegWHI. Passive procedural and distributive justice were also found to be directly and negatively related to NegWHI. We used the bootstrapping technique to estimate indirect effects (Preacher & Hayes, 2008). As shown in Table 4, the indirect effects of OJ on NegWHI through POS were all significant. Thus, POS totally mediates the effects of active procedural, informational and interpersonal justice on NegWHI, and partially mediates the effects of passive procedural and distributive justice on NegWHI. These findings partially support Hypothesis 1.

**Table 4:** Indirect pathways using bootstrapping (hypotheses 1 and 2). *Note*. *N* = 509. NegWHI = negative work-home interference; 10,000 bootstrap samples.

Indirect effect: x → m → y	Bootstrapping		Percentile 95% CI	

	Effect	SE	Lower	Upper

Active procedural justice → Perceived organizational support → NegWHI	−.0125	.0058	−.0274	−.0036
Passive procedural justice → Perceived organizational support → NegWHI	−.0225	.0099	−.0481	−.0077
Distributive justice → Perceived organizational support → NegWHI	−.0211	.0097	−.0453	−.0065
Interpersonal justice → Perceived organizational support → NegWHI	−.0155	.0095	−.0388	−.0009
Informational justice → Perceived organizational support → NegWHI	−.0263	.0121	−.0564	−.0077

Perceived organizational support → NegWHI → Job engagement	.0093	.0070	−.0019	.0263
Perceived organizational support → NegWHI → Job strain	−.0848	.0256	−.1375	−.0374
Perceived organizational support → NegWHI → Job satisfaction	.0858	.0280	.0368	.1477
Perceived organizational support à NegWHI à Intention to quit	−.0782	.0264	−.1368	−.0331

Concerning our second hypothesis, results showed that NegWHI is positively related to job strain and intention to quit and negatively related to job satisfaction. NegWHI has no significant relation with job engagement. POS was directly and positively related to job engagement and job satisfaction, and negatively related to intention to quit. As shown in Table 4, the indirect effects of POS on outcomes through NegWHI were significant only for job strain, job satisfaction and intention to quit. NegWHI totally mediates the effect of POS on job strain, and partially mediates the effects of POS on job satisfaction and intention to quit. These results partially support Hypothesis 2.

Ancillary analyses were conducted in order to investigate whether POS and NegWHI sequentially mediate the relationship between OJ and the outcomes. As indicated in Table 5, the indirect effects of the five dimensions of OJ on job strain, job satisfaction and intention to quit through POS and NegWHI were statistically different from zero. In sum, there is a double mediation in sequence but only for three of our outcomes.

**Table 5:** Indirect pathways using bootstrapping (Ancillary analyses: double mediation). *Note*. *N* = 509. POS = perceived organizational support; NegWHI = negative work-home interference; SE = Standard Error; CI=Confidence Interval; 10,000 bootstrap samples.

Indirect effect: x → m1 → m2 → y	Bootstrapping		Percentile 95% CI	

	Effect	SE	Lower	Upper

Active procedural justice → POS → NegWHI → Job engagement	.0008	.0007	−.0001	.0027
Active procedural justice → POS → NegWHI → Job strain	−.0069	.0033	−.0153	−.0019
Active procedural justice → POS → NegWHI → Job satisfaction	.0069	.0034	.0020	.0162
Active procedural justice → POS → NegWHI → Intention to quit	−.0063	.0032	−.0151	−.0018

Passive procedural justice → POS → NegWHI → Job engagement	.0014	.0011	−.0001	.0049
Passive procedural justice → POS → NegWHI → Job strain	−.0124	.0056	−.0264	−.0039
Passive procedural justice → POS → NegWHI → Job satisfaction	.0125	.0058	.0042	.0276
Passive procedural justice → POS → NegWHI → Intention to quit	−.0114	.0055	−.0269	−.0036

Distributive justice → POS → NegWHI → Job engagement	.0013	.0011	−.0001	.0047
Distributive justice → POS → NegWHI → Job strain	−.0117	.0056	−.0255	−.0033
Distributive justice → POS → NegWHI → Job satisfaction	.0118	.0058	.0036	.0271
Distributive justice → POS → NegWHI → Intention to quit	−.0108	.0054	−.0248	−.0030

Interpersonal justice → POS → NegWHI → Job engagement	.0009	.0009	−.0001	.0041
Interpersonal justice → POS → NegWHI → Job strain	-.0085	.0055	−.0225	−.0004
Interpersonal justice → POS → NegWHI → Job satisfaction	.0086	.0056	.0007	.0236
Interpersonal justice → POS → NegWHI → Intention to quit	−.0079	.0051	−.0217	−.0003

Informational justice → POS → NegWHI → Job engagement	.0016	.0014	−.0001	.0059
Informational justice → POS → NegWHI → Job strain	−.0145	.0068	−.0326	−.0045
Informational justice → POS → NegWHI → Job satisfaction	.0147	.0070	.0046	.0340
Informational justice → POS → NegWHI → Intention to quit	−.0134	.0066	−.0318	−.0042

## Discussion and conclusions

Previous studies analyzing the OJ-NegWHI relationship have shown that perceiving fairness at work facilitates the balance between work and family responsibilities (e.g., Lambert et al., 2013). However, these studies do not explain why OJ reduces the perception of NegWHI. In order to understand the underlying mechanism, the fist aim of this study is to consider the OJ-NegWHI relationship from a social exchange perspective. To the best of our knowledge, this study is the first to do so. Our first hypothesis postulated that POS mediates the negative relationship between OJ and NegWHI. The inclusion of these variables provides important implications for expanding our understanding of work-life balance in organizations. Based on the bootstrapping method, our findings demonstrate the indirect effect of perceiving fairness in work-family conciliation, on the perception of NegWHI through POS. Therefore, our results provided support for our first hypothesis. By perceiving fairness in work-family conciliation, workers feel supported by their organization and this support is, in turn, negatively related to their perception of NegWHI. Our study thus shows the importance of both OJ and POS in employees’ perception of NegWHI. These results are consistent both with research studying the relationship between OJ and POS on the one hand, and the relationship between POS and NegWHI on the other hand (e.g., Cheung, 2013; Wadsworth & Owens, 2007). Fairness in the workplace, and notably in work-family conciliation policies, is viewed by employees as evidence that the organization cares about their well-being and values their contributions (Rhoades & Eisenberger, 2002). By knowing that help is available and by enhancing perceived competence, employees who feel supported by their organization perceive themselves to have more resources to manage work and family demands (Rhoades & Eisenberger, 2002). Distributive and passive procedural justices are additionally directly associated with NegWHI. These results are consistent with previous research. When workers perceive that work-family conciliation strategies do not match with their contributions (i.e., distributive justice), they experience a poor fit between themselves and their environment (e.g., Kristof, 1996). This situation generates negative emotions which can spill over and negatively affect individuals’ functioning at home (Lambert, 2003). Likewise, when workers perceive that these strategies are based on biased information or violate ethical and moral standards (i.e., passive procedural justice), they experience negative feelings that increase the risk of feeling conflict at home. Our results show that these negative emotions have an influence on NegWHI. Indeed, in a context of an unfair process, individuals perceive higher negative emotions than in a context of a fair process (e.g., Krehbiel & Cropanzano, 2000). Therefore, our study shows that POS is a partial mediator in the relationship between two forms of OJ (i.e., distributive and passive procedural justices) and NegWHI; and is a total mediator in the relationship between the three other forms of OJ and NegWHI.

Previous studies have examined the effect of POS on employees’ outcomes by investigating several mechanisms such as commitment (e.g., Maertz et al., 2007) or self-determined work motivation (e.g., Gillet, Huart, et al., 2013). To expand the understanding of this relationship, we consider the mediating role of NegWHI. Therefore, the second hypothesis of our study postulated that NegWHI mediates the effects of POS on four employee outcomes (i.e., job strain, intention to quit, job satisfaction and job engagement). Based on the bootstrapping method, our findings demonstrate the indirect effect of perceiving support from the organization on only three of these employee outcomes (i.e., job strain, intention to quit and job satisfaction) through NegWHI. Consequently, these results partially support our second assumption. By feeling supported by the organization, workers perceive less negative interference between their private and professional lives and this interference is, in turn, positively related to job strain, intention to quit and negatively related to job satisfaction. These results are consistent with research studying the relationship between POS and NegWHI as well as with that studying the relationship between NegWHI and these three employee outcomes. Feeling supported is positively related to workers’ sense of being able to cope more effectively with their various roles (Rhoades & Eisenberger, 2002), leading them to experience less NegWHI. Trying to meet all the responsibilities of social roles drains individuals’ limited resources (Edwards & Rothbard, 2000), negatively impacting role enactment and causing job strain (Aryee et al., 2005). In order to protect their limited resources (Hobfoll, 1989), workers perceiving NegWHI have greater intention to quit their organization (Greenhaus et al., 1997). Individuals perceiving difficulties in fulfilling family responsibilities are not able to attain their values at work and this leads them to be less satisfied with their job (Perrewe et al., 1999). Results also show that POS is additionally directly associated with job satisfaction and intention to quit. By indicating that resources are available when needed, POS directly increases job satisfaction (e.g., Gillet, Colombat, et al., 2013). Because they feel obliged to return the favorable treatment they received (Gouldner, 1960), employees perceiving support express stronger feelings of affiliation and loyalty toward their organization and thus are more inclined to stay (e.g., Dawley et al., 2010). Therefore, our study shows that NegWHI is a partial mediator in the relationship between POS and two employee outcomes (i.e., intention to quit and job satisfaction); and it is a total mediator in the relationship between POS and job strain.

While testing our theoretical model, the NegWHI-job engagement relationship was significantly negative. When individuals perceive NegWHI due to a drain on or a threat to resources, they engage less in order to protect these resources. Indeed, according to the conservation of resources theory (Hobfoll, 1989), basic human motivation is directed toward the creation, maintenance, protection and accumulation of resources. However, this link became non-significant in our final model when we added a direct path from POS to job engagement. It is largely recognized that the more job resources (such as support) are available, the more employees feel engaged. Indeed, in line with the motivational process of the job demands-resources model (Bakker & Demerouti, 2008), job resources play an intrinsic and an extrinsic motivational role leading to basic needs fulfilment and to a willingness to invest effort in work. The non-significant NegWHI-job engagement relationship found in our final model might possibly be explained by the very low level of NegWHI experienced by the respondents of our study (i.e., mean of 1.01, range = 0–3). Indeed, Wilczek-Ruzyczka et al.’s (2012) found that this negative relationship occurs when NegWHI is experienced at a higher level. Possibly, within a sample perceiving more NegWHI, the NegWHI-job engagement relationship would have remained significant. This issue should be investigated in future studies.

### Limitations and perspectives for future research

This study is not without limitations and the findings reported here should be interpreted with caution. The major limitation is that our research design is cross-sectional, which precludes any inference of causality among the variables. Therefore, future studies should aim at replicating the findings of the present study by using longitudinal designs with repeated measures to confirm the direction of causality in our model. Due to the use of self-reported data, common-method variance may have biased our results (Podsakoff, MacKenzie, & Podsakoff, 2012). Nevertheless, we performed the Harman single-factor test and results indicated that the common method bias was not a major threat to our results. As our data were collected in the health care profession, it is also difficult to generalize our results to other professional sectors. Therefore, we encourage future studies to replicate this study with other samples.

Given its influence on a variety of work attitudes and behaviors, we focused only on the negative side of work-home interference. Indeed, we find it important to identify the determinants of NegWHI in the interests of both organizational effectiveness and employee well-being. However, in view of emerging research concerning the positive side of work-home interference (referring to the fact that work provides resource gains that enhance one’s performance in the family domain; Greenhaus & Powell, 2006), it would be interesting to investigate both positive and negative aspects. Indeed, several authors recommended integrating positive work-home interference into research on work-family balance (e.g., Carlson, Kacmar, Wayne, & Grzywacz, 2006). According to Lourel and St-Onge (2012), if conflict provides a partial explanation of reality, it is also important to study the enriching or facilitating character of work-family interference.

Studies exploring the concept of overall justice have increased (e.g., Ambrose & Arnaud, 2005) in recent years. Through their study, Nicklin, McNall, Cerasoli, Strahan, and Cavanaugh (2014) examined the role of overall justice within Colquitt’s (2001) four-dimensional framework. They found that overall justice was best conceptualized as a unique independent construct, predicted from (but not an average of) the four justice dimensions. Thus, their results indicate that overall justice is not a second-order hierarchical construct. These authors recommend continuing to examine the interplay between the specific justice dimensions and overall justice in order to predict organizational attitudes and outcomes. Therefore, future research could replicate our study by integrating the concept of overall justice to test, for example, its mediating role between specific justice dimensions and POS.

### Practical implications

Considering the negative relationships between NegWHI and several outcomes, institutions should pay close attention to NegWHI. This study offers organizations some suggestions how NegWHI can be used to fulfill employees’ needs.

Organizations could help individuals to better manage their work and family lives and reduce the impact of NegWHI by enhancing employees’ feelings of being supported by their organization. To increase these feelings, managers could for example, communicate the voluntary nature of favorable actions and the involuntary nature of unfavorable ones; display sincerity through consistency of discourse and actions; treat employees fairly, respectfully and courteously; provide meaningful training and developmental programs promoting personal growth, knowledge and career goals and also promote fairness in administering policies and allocating rewards (Eisenberger & Stinglhamber, 2011). Support groups made up of peers could also be proposed to analyze difficult job situations together, to express problems and to eliminate isolation (e.g., Balint groups; Johnson, Nease, Milberg, & Addison, 2004).

Work-family conciliation policies and strategies have to be fair and well-established, notably by taking account of employees’ opinions, by being applied appropriately based on accurate or unbiased information and by matching employees’ contributions. Employees have to receive appropriate and precise information, and be treated with dignity and respect in the administration of these policies.

## Competing interests

The authors declare that they have no competing interests.
